# Circulating microRNA Profiles in Patients with Type-1 Autoimmune Hepatitis

**DOI:** 10.1371/journal.pone.0136908

**Published:** 2015-11-17

**Authors:** Kiyoshi Migita, Atsumasa Komori, Hideko Kozuru, Yuka Jiuchi, Minoru Nakamura, Michio Yasunami, Hiroshi Furukawa, Seigo Abiru, Kazumi Yamasaki, Shinya Nagaoka, Satoru Hashimoto, Shigemune Bekki, Hiroshi Kamitsukasa, Yoko Nakamura, Hajime Ohta, Masaaki Shimada, Hironao Takahashi, Eiji Mita, Taizo Hijioka, Haruhiro Yamashita, Hiroshi Kouno, Makoto Nakamuta, Keisuke Ario, Toyokichi Muro, Hironori Sakai, Kazuhiro Sugi, Hideo Nishimura, Kaname Yoshizawa, Takeaki Sato, Atsushi Naganuma, Tatsuji Komatsu, Yukio Oohara, Fujio Makita, Minoru Tomizawa, Hiroshi Yatsuhashi

**Affiliations:** 1 NHO-AIH study group, Nagasaki Medical Center, kubara 2-1001-1 Omura, Nagasaki, 856–8562, Japan; 2 Department of Hepatology, Nagasaki University Graduate School of Biomedical Sciences, Nagasaki University, 1-7-1 Sakamoto, Nagasaki, Japan; 3 Department of Clinical Medicine, Institute of Tropical Medicine, Nagasaki University, Sakamoto 1-7-1, Nagasaki, 852–8501, Japan; 4 Molecular and Genetic Epidemiology Laboratory, Faculty of Medicine, University of Tsukuba, 1-1-1 Tennodai, Tsukuba, 305–8575, Japan; SAINT LOUIS UNIVERSITY, UNITED STATES

## Abstract

Recent studies have demonstrated that micro (mi)RNA molecules can be detected in the circulation and can serve as potential biomarkers of various diseases. This study used microarray analysis to identify aberrantly expressed circulating miRNAs in patients with type 1 autoimmune hepatitis (AIH) compared with healthy controls. Patients with well-documented and untreated AIH were selected from the National Hospital Organization (NHO)-AIH-liver-network database. They underwent blood sampling and liver biopsy with inflammation grading and fibrosis staging before receiving treatment. To further confirm the microarray data, circulating expression levels of miR-21 and miR-122 were quantified by real-time quantitative polymerase chain reaction in 46 AIH patients, 40 patients with chronic hepatitis C (CHC), and 13 healthy controls. Consistent with the microarray data, serum levels of miR-21 were significantly elevated in AIH patients compared with CHC patients and healthy controls. miR-21 and miR-122 serum levels correlated with alanine aminotransferase levels. Circulating levels of miR-21 and miR-122 were significantly reduced in AIH patients with liver cirrhosis, and were inversely correlated with increased stages of fibrosis. By contrast, levels of circulating miR-21 showed a significant correlation with the histological grades of inflammation in AIH. We postulate that aberrantly expressed serum miRNAs are potential biomarkers of AIH and could be implicated in AIH pathogenesis. Alternations of miR-21 and miR-122 serum levels could reflect their putative roles in the mediation of inflammatory processes in AIH.

## Introduction

Micro RNAs (miRNAs) are small endogenous RNA molecules of 19–24 nucleotides that control the translation and transcription of targeting RNAs by base-pairing to complementary sites [1]. Serum miRNA expression is stable, reproducible, and consistent among individuals of the same species, and specific expression patterns have been identified as biomarkers for numerous diseases and cancers [2]. For example, miR-122, the most abundant miRNA in hepatocytes, has a well-defined role in hepatitis C virus (HCV) replication, and serves as a viable therapeutic target [3]. A role for miR-122 is also emerging in other liver diseases [4].

Ample evidence exists for the important regulatory potential of other miRNAs in conditions associated with liver inflammation, the metabolic syndrome, or autoimmune processes [5]. miRNAs regulate the function of both the innate and the adaptive immune system, and altered miRNA expression has been reported in human autoimmune diseases [6]. For instance, a unique miRNA expression profile was demonstrated in the sera from an individual with the autoimmune liver disease primary biliary cirrhosis [7].

Autoimmune hepatitis (AIH) is a rare disease characterized by hypergammaglobulinemia, the production of autoantibodies and a good response to immunosuppression [8]. Different subtypes of AIH may be distinguished from differences in the autoantibody patterns. For example, AIH type 1 is characterized by anti-nuclear antibodies (ANA) and/or anti-smooth muscular antibodies (SMA) [9]. The etiology of AIH is unknown, but it is thought to have both a genetic and an environmental basis [10]. miRNAs are emerging as highly tissue-specific biomarkers with the potential applicability to revolutionize disease diagnosis and treatment [11]. In this study, therefore, we used microarray for the initial screening followed by quantitative reverse transcription PCR (qRT-PCR) validation to analyze serum samples from patients with type I AIH. Our results demonstrated that the unique expression pattern of serum miRNAs can serve as a non-invasive biomarker for AIH.

## Materials and Methods

### Study population

Patients with well-documented and untreated AIH were enrolled from the National Hospital Organization (NHO)-AIH-liver-network database, a multicenter registry for Japanese patients with AIH [12]. Almost patients were enrolled in Nagasaki Medical Center and Nagoya Medical Center. All patients satisfied the 1999 revised criteria of International Autoimmune Hepatitis Group diagnosis of type-1 AIH [13]. Patients were excluded from the study if there was histological evidence of cholangitis or non-alcoholic steatohepatitis. Patients positive for hepatitis B virus surface antigen or HCV RNA were also excluded. Patients with other causes of liver disease, such as excess alcohol or drug use, were excluded based on reviews of their appropriate history and investigations. As controls, patients with chronic hepatitis C (CHC) (n = 40; female/male = 20/20; genotype 1b:29, 2a:7, 2b:4; mean age, 60.5±9.3 years; aspartate aminotransferase (AST), 76.0±63.0 IU/L; alanine transaminase (ALT), 94.5±96.8 IU/L), and healthy controls (n = 13; female/male = 7/6; mean age, 42.5±14.4 years) were included. The study was approved by the Ethics Committee of the NHO Central Internal Review Board and participating NHO liver-network hospitals. Written informed consent was obtained from each individual.

### Clinical and histological assessments

Serum concentrations of type IV collagen were determined with a commercial RIA kit (Panassay IV. C, Daiichi Chemical Co. Ltd. Tokyo, Japan).

Liver biopsy and laboratory tests were obtained at baseline prior to treatment. In the histological diagnosis of AIH, each specimen was assessed for inflammatory grading including the degree of portal inflammation, presence of interface hepatitis, and the degree of parenchymal inflammation, as well as the stage of fibrosis (0, absent; 1, expansion of fibrosis to parenchyma; 2, portal–central or portal–portal bridging fibrosis; 3, presence of numerous fibrous septa; and 4, multi-nodular cirrhosis) according to the criteria of Desmet et al. [14]. Cirrhosis was diagnosed histologically when a loss of normal lobular architecture, reconstruction of hepatic nodules, and the presence of regenerative nodules were observed.

### Microarray analysis of serum miRNAs

We prepared a serum pool from five patients with untreated with acute-onset type AIH and stocked paired sera before/after glucocorticoid treatments. A control miRNA pool from five healthy subjects. RNA was isolated from serum samples using QIAzol reagent according to the manufacturer’s instructions (Qiagen, Hilden, Germany). Microarray analysis was performed to evaluate miRNA expression patterns in serum pools from patients and controls using 3D-Gene miRNA Oligo chips according to the manufacturer’s instructions (Toray Industries, Inc., Tokyo, Japan). Small RNAs from serum were labelled using the miRCURY LNA microRNA Array Power Labelling Kit (Exiqon. Palm Beach FL, USA) and analyzed using 3D-Gene miRNA Oligo chips Ver. 17.0 (Toray Industries Inc.) containing more than 1700 antisense probes printed in duplicate spots, according to the manufacturer’s instructions. Signals were analyzed using the 3D-Gene Scanner 3000 (Toray Industries Inc. Tokyo, Japan). microRNAs array data from this study are in agreement with the Minimum Information About a Microarray Experiment (MIAME) and are publicly available through the Gene Expression Omnibus (GEO) database (http://www.ncbi.nlm.nih.gov/projects/geo/) under the accession number GSE71432.

### Quantitative reverse transcription PCR validation study

More serum samples of AIH patients (n = 46), CHC patients (n = 40), and healthy controls (n = 13) were used in a qRT-PCR validation study. We followed the protocol previously reported by Mitchell et al. [15] to determine endogenous levels of *Caenorhabditis elegans* miR-39 miRNA (miRNeasy Serum/plasma Spike-In Control). RNA was isolated from serum samples using Qiazol reagent according to the manufacturer’s instructions (Qiagen, Limburg, the Netherlands). *C*. *elegans* miR-39 (Qiagen) was added to serum samples (1.6 ×10^8^ copies/μl) prior to the RNA isolation procedure for the later normalization of extracellular miR-122 and miR-21 levels. The RNA quality was assessed by microcapillary electrophoresis (2100 BioAnalyser, Agilent Technologies, Waldbronn, Germany).

cDNA was reverse transcribed from 2.5 μl RNA using the TaqMan miRNA reverse transcription kit. qRT-PCR for the detection of hsa-miR-21 and miR-122 was carried out in 20 μl PCR reactions using the TaqMan MicroRNA assay with the StepOnePlus detection system (Applied Biosystems) at 50°C for 2 min then 95°C for 10 min, followed by 40 cycles of 95°C for 15 s and 60°C for 1 min.

### Statistical methods

Expression levels of selected miRNAs detected by qRT-PCR were normalized to cel-miR-39 and presented as the fold-change (2−ΔΔCt) above the control (Control-1): ΔΔCt = (CtmiRNA-Ctcel-miR-39) patient mean − (CtmiRNA-Ctcel-miR-39) control mean. Results for non-normally distributed continuous variables were summarized as medians (interquartile ranges) and were compared by the Mann-Whitney U test. Paired data were analyzed by non-parametric tests using the Wilcoxon signed-rank test for the comparison of paired data. Correlation coefficients (r) were calculated using the Spearman correlation.

## Results

### Demographic data of AIH patients

Table 1 shows demographic data of AIH patents. Among the 46 patients with type-1 AIH, 31 (67.4%) were positive for ANA (>1:40) and 32 (69.6%) for SMA (>1:40). Nine (19.6%) had liver cirrhosis at the time of diagnosis.

**Table 1:** Baseline Characteristics of 46 Japanese AIH Type 1 Patients. AST, aspartate aminotransferase; ALT, alanine aminotransferase; ALP, alkaline phosphate; IgG, immunoglobulin G; ANA, anti-nuclear antibody; ASMA, anti-smooth muscle antibody; IQR, interquartile range; IAIHG, International Autoimmune Hepatitis Group.

Characteristics	n = 46
Female, n/total (%)	37/46(80.4)
Age, y, mean ± SD	59.6 ± 12.8
Biochemistry	
AST, IU/L, median (IQR)	290.5(97–724)
ALT, IU/L, median (IQR)	405.5(115–842)
ALP, IU/L, median (IQR)	437.5(347–514)
Total Bilirubin, mg/dl, median (IQR)	1.3(0.9–3.3)
Albumin, g/dl, median (IQR)	3.9(3.5–4.2)
IgG, mg/dl, median (IQR)	1860.0(1584–2370)
Prothrombin time, %, median (IQR)	81.3(72.3–89.0)
Platelets, 10^4^/μl, median (IQR)	18.0(14.4–21.2)
Serology	
ANA ≧1:40, n/total (%)	31/46(67.4)
ASMA ≧1:40, n/total (%)	32/46(69.6)
Histology	
Cirrhosis, n/total (%)	9/46(19.6)
IAIHG criteria	
Score, median (IQR)	17(15–18)

### Unique miRNA expression pattern in AIH

We used the microarray system to determine those circulating miRNAs that were differentially expressed in AIH patients enrolled in Nagasaki Medical Center, Nagoya Medical Center and Ueda Medical Center. Of 2,555 miRNAs assayed, 811 were expressed at greater than background levels. S1 Fig shows the miRNAs that were expressed at higher levels in the sera from AIH patients compared with healthy subjects. A comparison between AIH patients and controls identified 11 miRNAs that were up-regulated by more than 1.7-fold (Table 2). Furthermore, comparing before and after successful glucocorticoid therapy identified 10 miRNAs that were down-regulated by up to 0.4-fold (S1 Fig and Table 3). Among these isolated miRNAs, miR-21 and miR-122 showed similar expression profiles, and were specifically up-regulated in untreated AIH patients and down-regulated in the remission phase after corticosteroid therapy.

**Table 2:** Differentially expression miRNAs between control and AIH.

up			down		
no.	miR_name	fold change	no.	miR_name	fold change
1	hsa-miR-122-5p	7.34	1	hsa-miR-223-3p	0.14
2	hsa-miR-1915-5p	2.99	2	hsa-miR-575	0.16
3	hsa-miR-193b-3p	2.83	3	hsa-miR-451a	0.18
4	hsa-miR-1908-3p	2.72	4	hsa-miR-4638-5p	0.25
5	hsa-miR-6073	2.63	5	hsa-miR-4443	0.26
6	hsa-miR-99a-5p	2.62	6	hsa-miR-486-5p	0.28
7	hsa-miR-602	1.95	7	hsa-miR-6765-3p	0.29
8	hsa-miR-1199-5p	1.87	8	hsa-miR-6820-5p	0.29
9	hsa-miR-1290	1.78	9	hsa-miR-4648	0.30
10	hsa-miR-21-5p	1.77	10	hsa-miR-6511a-5p	0.32
11	hsa-miR-4732-5p	1.72	11	hsa-miR-6889-5p	0.37
			12	hsa-miR-1207-5p	0.38
			13	hsa-miR-7150	0.39
			14	hsa-miR-6877-5p	0.40
			15	hsa-miR-4476	0.42
			16	hsa-miR-6763-5p	0.43

**Table 3:** Differentially expression miRNAs between befor and after corticosteroid therapy.

up			down		
no.	miR_name	fold change	no.	miR_name	fold change
1	hsa-miR-23a-5p	3.60	1	hsa-miR-122-5p	0.01
2	hsa-miR-4450	3.15	2	hsa-miR-1290	0.14
3	hsa-miR-4294	2.92	3	hsa-miR-21-5p	0.15
4	hsa-miR-4478	2.87	4	hsa-miR-1246	0.15
5	hsa-miR-4733-3p	2.67	5	hsa-miR-4732-5p	0.18
6	hsa-miR-204-3p	2.64	6	hsa-miR-6073	0.20
7	hsa-miR-6076	2.60	7	hsa-miR-1908-3p	0.26
8	hsa-miR-4525	2.19	8	hsa-miR-602	0.30
9	hsa-miR-4665-5p	2.07	9	hsa-miR-92a-3p	0.33
10	hsa-miR-4769-5p	2.05	10	hsa-miR-1915-5p	0.34
11	hsa-miR-7641	1.92			
12	hsa-miR-4476	1.75			
13	hsa-miR-7150	1.75			
14	hsa-miR-6891-5p	1.65			
15	hsa-miR-7109-5p	1.59			
16	hsa-miR-6889-5p	1.52			

### Circulating levels of miR-21 and miR-122 in AIH and CHC patients

qRT-PCR was used to verify the data obtained from microarray analysis. Expression levels of miR-21 and miR-122 were normalized to spike-in cel-miR39 and were shown to be significantly elevated in AIH patients compared with both CHC patients and healthy subjects (S2 Fig).

### Reversibility of the increased expressed miRNA corticosteroid therapy

Circulating levels of miR-21 and miR-122 were determined before and after corticosteroid therapy in paired serum samples from AIH patients. miR-21 and miR-122 were both shown to be up-regulated in untreated AIH patients, and down-regulated following glucocorticoid treatment of these same patients (S3 Fig).

### Relationship between circulating miR-21 and miR-122 and liver function tests

To investigate the relationship between circulating miR-21 levels and standard liver function parameters, we examined correlations between circulating miR-21 or miR-122 and serum levels of AST or ALT (S4 Fig). A positive correlation was observed between serum miR-21 and miR-122 levels and AST (miR-21: r = 0.820, *P*<0.0001; miR-122: r = 0.628, *P*<0.0001) and ALT (miR-21: r = 0.761, *P*<0.0001; miR-122: r = 0.662, *P*<0.0001). However, the correlation rate was higher with respect to serum miR-21 compared with serum miR-122. We could not find a significant correlation between serum miR-21 or miR-122 levels and those of type IV collagen (S5 Fig).

### Relationship between circulating miR-21 and miR-122, liver fibrosis and necroinflammation

As shown in S6 Fig, AIH patients with liver cirrhosis (LC) had significantly lower levels of circulating miR-21 and miR-122 compared with those without LC. Moreover, serum miR-21 and miR-122 levels were also shown to be reduced in AIH patients with an advanced fibrosis stage (S7A Fig). To evaluate whether serum miR-21 and miR-122 were correlated with hepatic necroinflammation, we evaluated these circulating miRNAs in AIH patients sub grouped according to the grading of necroinflammation. Liver necroinflammation grading correlated positively and significantly with circulating levels of miR-21, but not with miR-122 (S7B Fig).

## Discussion

miRNA changes in the liver have been reported in diseases such as viral hepatitis and hepatocellular carcinoma (HCC) [16, 17]. However, little is known about their detection in the blood from AIH patients. The current study provides the first evidence that AIH is associated with altered circulating miRNA expression. We demonstrated that circulating has-miR-21 and miR-122 were significantly elevated in patients with AIH leading to a unique miRNA expression profile, but not in those with AIH combined with LC. We also detected strong correlations between serum miR-21 and miR-122 levels and ALT, a parameter of ongoing liver damage, and a negative correlation with liver fibrosis scores. The persistent liver damage responsible for perpetuating this process is characterised by the infiltration of immune cells into the liver and the death of hepatocytes. miRNAs are essential for the regulation of liver development, regeneration, and metabolic functions [18]. Hence, alternations in intrahepatic miRNA networks are associated with liver diseases, including hepatitis, steatosis, cirrhosis, and HCC. miR-122 is the most common miRNA with aberrant expression identified in liver disease, serving as a biomarker of liver injury in chronic hepatitis B or C, non-alcoholic fatty-liver disease, and drug-induced liver disease [19–22]. It is also an essential host factor for HCV infection and an antiviral target [23]. Circulating miR-122 levels were previously shown to be massively increased in response to hepatic injury and intrahepatic loss of miR-122 [24]. This loss was not significantly correlated with grades of inflammation in CHC infection, although it was significantly correlated with fibrosis in affected patients [25]. Our findings support previous studies demonstrating liver-specific loss of miR-122 at late stages of fibrosis in CHC infection. However, our data showed that circulating miR-21 levels were significantly positively associated with grades of inflammation and negatively with fibrosis.

Haider et al. performed the global evaluation of miRNAs as biomarkers for non-neoplastic diseases [26]. They isolated six potential miRNA biomarkers of hepatic injury, of which only miR-122 was demonstrated to be liver-specific [26]. miR-122 significantly increased type I interferon (IFN) expression in hepatocytes, presumably through modulation of suppressor of cytokine signaling 1expression [27]. These findings suggest that miR-122 may also contribute to autoimmunity in liver through the IFN-signaling pathway. It has also been demonstrated that miR-21 is induced after hepatectomy and contributes to liver regeneration by inducing the translation of cyclin D [28]. These data suggest that the higher miR-21 serum levels observed in AIH have similar functions to those seen after acute liver injury and following liver regeneration.

Mechanistically, it is possible for miR-21 to leak from damaged cells similar to ALT. These circulating levels are negatively regulated by hepatic fibrosis, as shown by the correlation between miR-21 and hepatic fibrosis. Our data are in line with these findings, indicating that serum miR-21 levels are related to liver damage activity rather than liver fibrosis in patients with CHC [29]. Thus, miR-21 and miR-122 may both originate from the inflamed liver in AIH, although miR-21, but not miR122, correlates with total bilirubin and the grading of liver inflammation. Therefore, serum levels of miR-21 and miR-122 reflect overlapping, but not identical, disease parameters in AIH patients. This might be related to the differential expression patterns of miR-21 and miR-122, with miR-122 being highly selective for the liver [30], whereas miR-21 shows strong expression in other cells such as lymphocytes [31]. Hepatic immune cell infiltrations or activation may contribute to the elevation of serum miR-21 levels in patients with AIH. miRNAs are crucial to many aspects of immunity, including T-cell immunity [32], so we speculate that circulating miR-21 mirrors the presence of immune-mediated hepatocyte injury in patients with AIH.

miR-21 plays a crucial role in T cells by sustaining the proliferation and repression of apoptosis. It has also been reported to target tumor suppressor genes such as *Btf2*, *PDCD4*, *Pten*, and sprout, which regulate cell death and proliferation [33]. miR21 directly down-regulates the expression of *PDCD4*, which encodes a protein that localizes to the nucleus and plays a role in pathogenic T cell apoptosis and cell proliferation [34, 35]. This leads to acquisition of an activated phenotype in normal T cells following miRNA-21 overexpression. miR-21 regulates aberrant T cell responses through control of *PDCD4* expression in human systemic lupus erythematosus [36], suggesting that miR-21 induction enables T cells to elude apoptosis and enhance the secretion of pro-inflammatory cytokines such as interferon-γ and interleukin-17 by repressing *PDCD4* expression [37].

More recently, it was demonstrated that the miR-21 pathway intrinsically controls Th17 differentiation. CD4^+^ T cells lacking miR-21 were shown to exhibit a specific Th17 cell defect affecting the transforming growth factor-β pathway during experimental autoimmune encephalomyelitis *in vivo* [37]. Given the ameliorating effect of ant-miR-21 observed in autoimmune model mice, the increased circulating miR-21 demonstrated in AIH patients in the present study could play a critical role in immune-mediated liver injury, which could be a contributing factor in the development of AIH. Recently, a report demonstrated that AP-1 is essential for miR-21 expression because mutations in AP-1 binding sites eliminated miR-21 promoter activity [38]. Moreover, glucocorticoid is thought to regulate miR-21 transcription through inhibiting the action of proinflammatory transcription factors such as AP-I [39].

The present study has a number of limitations that should be considered when interpreting the results. First, we could not assess the relationship between the circulating levels of candidate miRNAs and fluctuations of lupus disease activity with time. Second, because of the unknown impact of factors that regulate circulating miRNA levels, we were unable to normalize for the miRNA content using a reliable housekeeping miRNA. However, we supplemented the samples with recombinant cel-lin-4, which can be consistently detected by qRT-PCR, and detected no significant differences in raw Ct values of cel-lin-4 among the three groups (AIH, CHC, and control); thus, cel-miR39 was used to normalize the difference in the efficiency of RNA isolation. Additionally our findings may not reflect other populations because we only focused on Japanese individuals. Hence, the sampling of other ethnic groups may result in a different circulating miRNA signature for AIH.

In conclusion, this is the first report to investigate the circulating miRNA profiles of AIH patients using microarray and quantitative real-time PCR analysis. We revealed that differential levels of serum miR-21 and miR-122 were associated with AIH, which may play important roles in disease pathogenesis. The mechanisms underlying the regulation of these aberrant circulating miRNAs remain to be investigated.

### NHO-AIH study group

Kiyoshi Migita, Yuka Jiuchi, Atsumasa Komori, Hiroshi Yatsuhashi, Minoru Nakamura (NHO Nagasaki Medical Center), Hiroshi Kamitsukasa (NHO Tokyo National Hospital), Yoko Nakamura (NHO Sagamihara National Hospital), Hajime Ohta (NHO Kanazawa Medical Center), Masaaki Shimada (NHO Nagoya Medical Center), Hironao Takahashi (NHO Higashi Nagoya National Hospital), Eiji Mita (Osaka National Hospital), Taizo Hijioka (NHO Osaka Minami Medical Center), Yabuuchi Iwao (NHO Minami Wakayama Medical Center), Haruhiro Yamashita (NHO Okayama Medical Center), Hiroshi Kouno (NHO Kure Medical Center), Noriaki Naeshiro (NHO Higashihiroshima Medical Center), Makoto Nakamuta (NHO Kyushu Medical Center), Keisuke Ario (NHO Ureshino Medical Center), Toyokichi Muro (NHO Oita Medical Center), Hironori Sakai (NHO Beppu Medical Center), Kazuhiro Sugi (NHO Kumamoto Medical Center), Hideo Nishimura (NHO Asahikawa Medical Center), Kaname Yoshizawa (NHO Shinshu Ueda Medical Center), Takeaki Sato (NHO Kokura Medical Center), Atsushi Naganuma (NHO Takasaki General Medical Center), Tatsuji Komatsu (NHO Yokohama Medical Center), Yukio Oohara (NHO Hokkaido Medical Center), Fujio Makita (NHO Nishigunma National Hospital), Minoru Tomizawa (NHO Shimoshizu National Hospital), Masahiro Kikuchi (NHO Tokyo Medical Center), Hiroshi Furukawa (University of Tsukuba Medical Medical molecular genetic epidemiology laboratory), Michio Yasunami (Institute of Tropical Medicine Nagasaki University).

## Supporting Information

S1 Fig Comparison of miRNA expressions in the sera from normal subjects and AIH patients by miRNA microarray. (A) Comparison of normalized signal intensities of various miRNAs in sera. X axis represents untreated AIH patients (n = 5) and Y axis represent healthy controls (n = 5). (B) Comparison of normalized signal intensities of various miRNAs in sera. X axis represents the sera from untreated (pretreatment) and Y axis represents the sera from after successful treatment (posttreatment) in the same AIH patients (n = 5). (GEO accession No:GSE71432)
(TIF)

S2 Fig Quantitative real-time PCR analysis for miR-21 and miR-122 in patients with AIH, chronic hepatitis C (CHC) and healthy subjects. The vertical lines indicate the range, horizontal boundaries of the boxes represent the first and third quartile. Results were compared by non-parametric Mann-Whitney test.
(TIFF)

S3 Fig Changes of serum miR-21 and miR-122 before and 4 weeks after the initiation of corticosteroid therapy. Thirty four AIH patients with paired serum samples (Before and 4 weeks after corticosteroid therapy) were subjected to qRT-PCR analysis for miR-122. The vertical lines indicate the range, horizontal boundaries of the boxes represent the first and third quartile. Paired samples from the same subjects were compared by Wilcoxon signed-rank test.
(TIFF)

S4 Fig Correlations between serum levels of miR-21or miR-122 and AST (A) or ALT (B). Correlations between serum levels of miR-21or miR-122 and serum AST levels were determined in patients with AIH. The correlation coefficient was determined by Pearson’s product statistic and the regression line is represented by the solid line.
(TIF)

S5 Fig Correlations between serum levels of miR-21or miR-122 and type IV collagen. Correlations between serum levels of miR-21or miR-122 and serum type IV collagen levels were determined in patients with AIH. The correlation coefficient was determined by Pearson’s product statistic and the regression line is represented by the solid line.
(TIF)

S6 Fig Serum levels of miR-21 or miR-122 in AIH patients with or without liver cirrhosis (LC). The vertical lines indicate the range, horizontal boundaries of the boxes represent the first and third quartile. Results were compared by non-parametric Mann-Whitney test.
(TIFF)

S7 Fig (A) Serum levels of miR-21 or miR-122(B) and relationship with stages of fibrosis. Correlations were assessed by Spearamn’s rank correlation. Serum levels of miR-21 and miR-122 correlated inversely and significantly with the stage of fibrosis. (miR-21, r = -0.309, *p* = 0.013, miR-122, r = -0.324, *p* = 0.009). (B) Serum levels of miR-21 or miR-122 and relationship with grades of necroinflammation. Correlations were assessed by Spearamn’s rank correlation. Serum levels of miR-21 correlated positively and significantly with the grade of inflammation. (r = 0.3385, *p* = 0.001). (TIF)
