# Supplementary figures and images for: Circulating microRNA Profiles in Patients with Type-1 Autoimmune Hepatitis
Source: PLoS One. 2015 Nov 17;10(11):e0136908. doi: 10.1371/journal.pone.0136908 (PMC4648542; doi:10.1371/journal.pone.0136908)

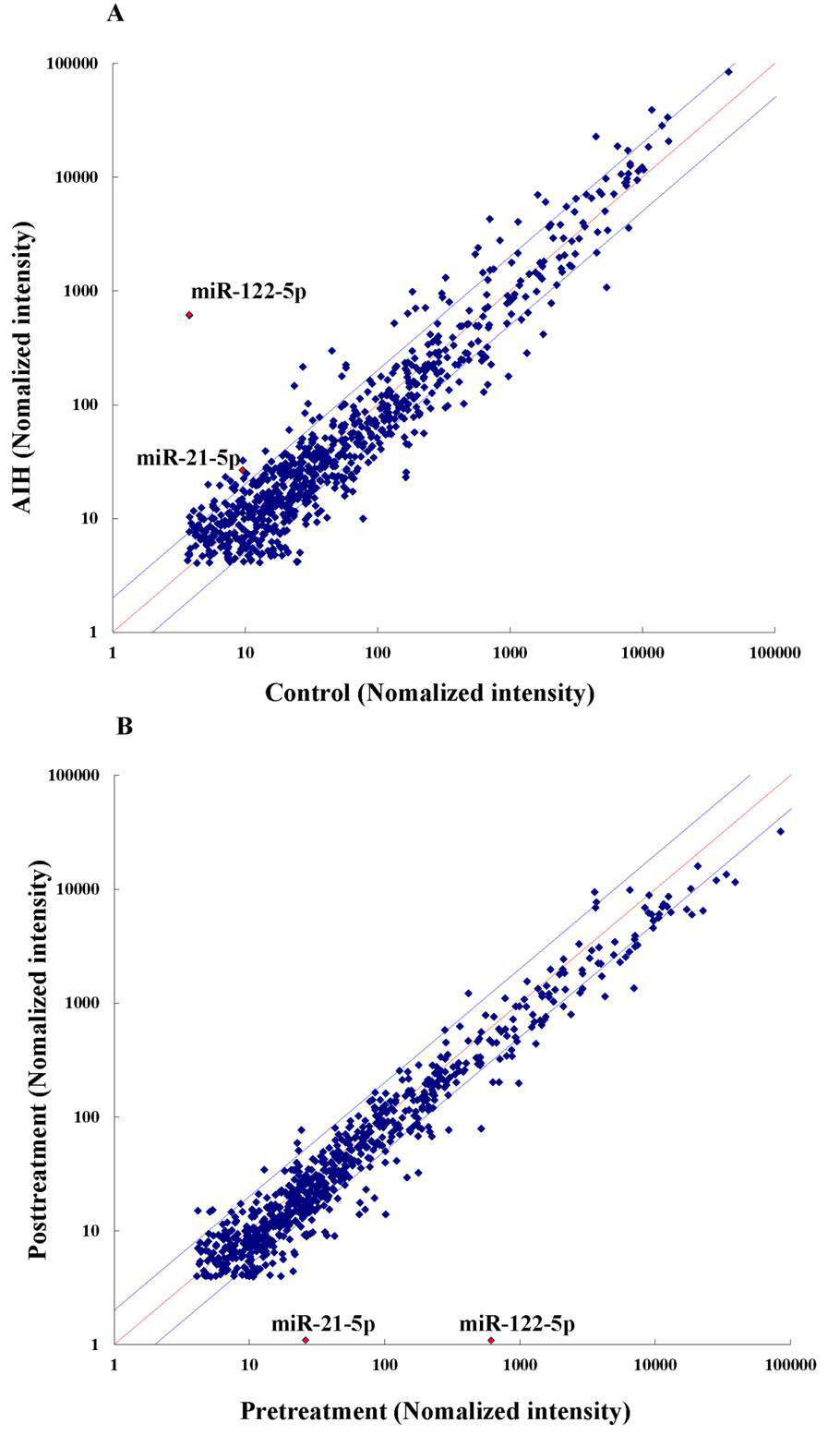

Supplement: S1 Fig — (A) Comparison of normalized signal intensities of various miRNAs in sera. X axis represents untreated AIH patients (n = 5) and Y axis represent healthy controls (n = 5). (B) Comparison of normalized signal intensities of various miRNAs in sera. X axis represents the sera from untreated (pretreatment) and Y axis represents the sera from after successful treatment (posttreatment) in the same AIH patients (n = 5). (GEO accession No:GSE71432) (TIF) [file pone.0136908.s001.tif]

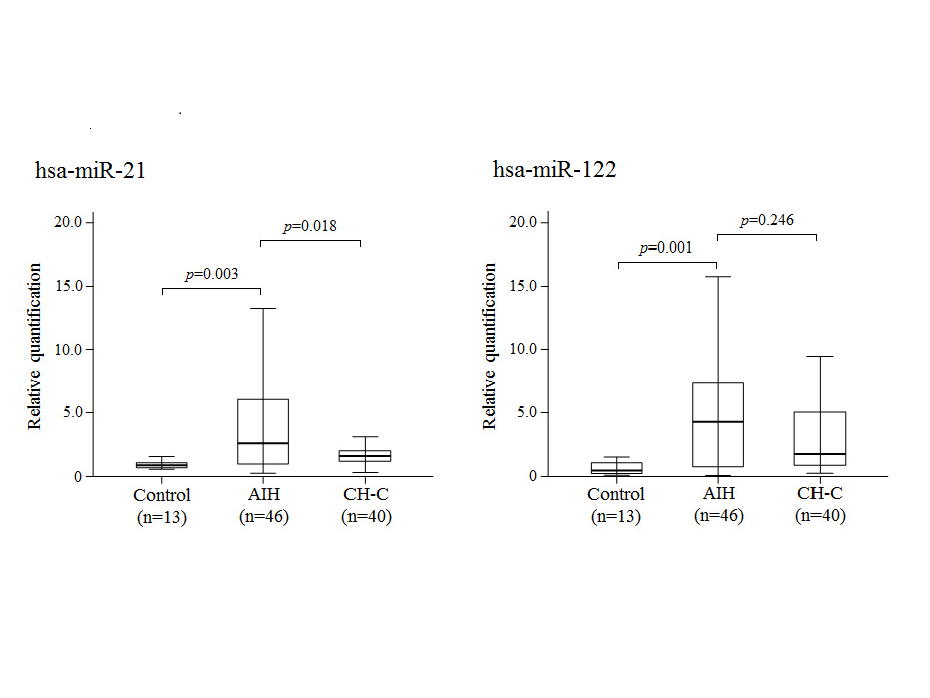

Supplement: S2 Fig — The vertical lines indicate the range, horizontal boundaries of the boxes represent the first and third quartile. Results were compared by non-parametric Mann-Whitney test. (TIFF) [file pone.0136908.s002.tiff]

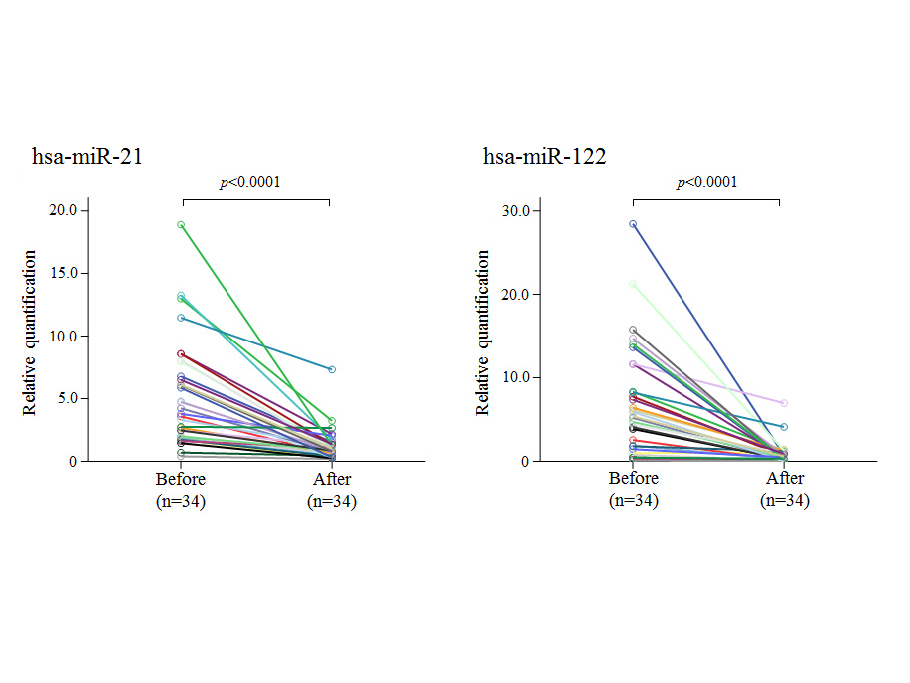

Supplement: S3 Fig — Thirty four AIH patients with paired serum samples (Before and 4 weeks after corticosteroid therapy) were subjected to qRT-PCR analysis for miR-122. The vertical lines indicate the range, horizontal boundaries of the boxes represent the first and third quartile. Paired samples from the same subjects were compared by Wilcoxon signed-rank test. (TIFF) [file pone.0136908.s003.tiff]

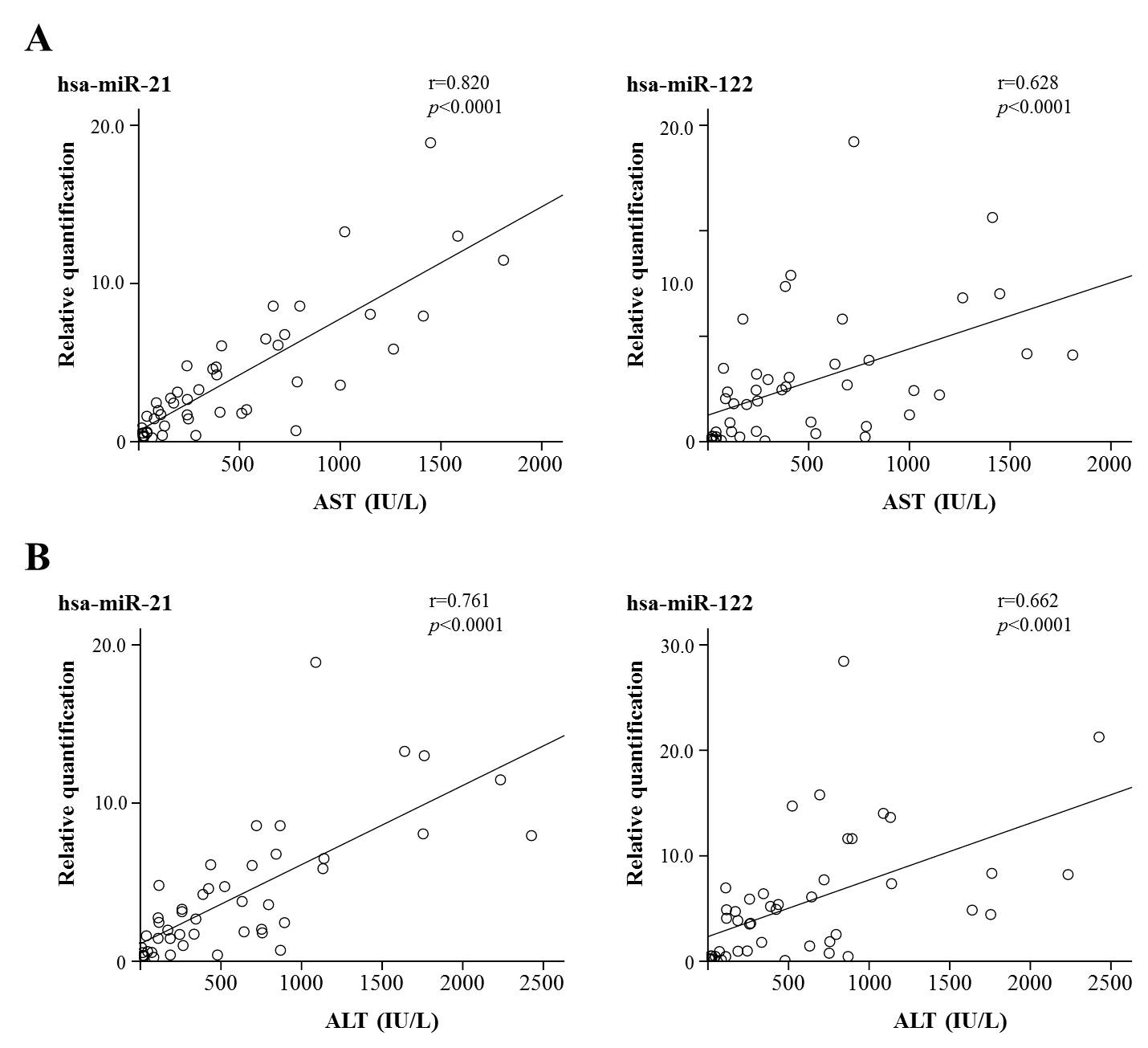

Supplement: S4 Fig — Correlations between serum levels of miR-21or miR-122 and serum AST levels were determined in patients with AIH. The correlation coefficient was determined by Pearson’s product statistic and the regression line is represented by the solid line. (TIF) [file pone.0136908.s004.tif]

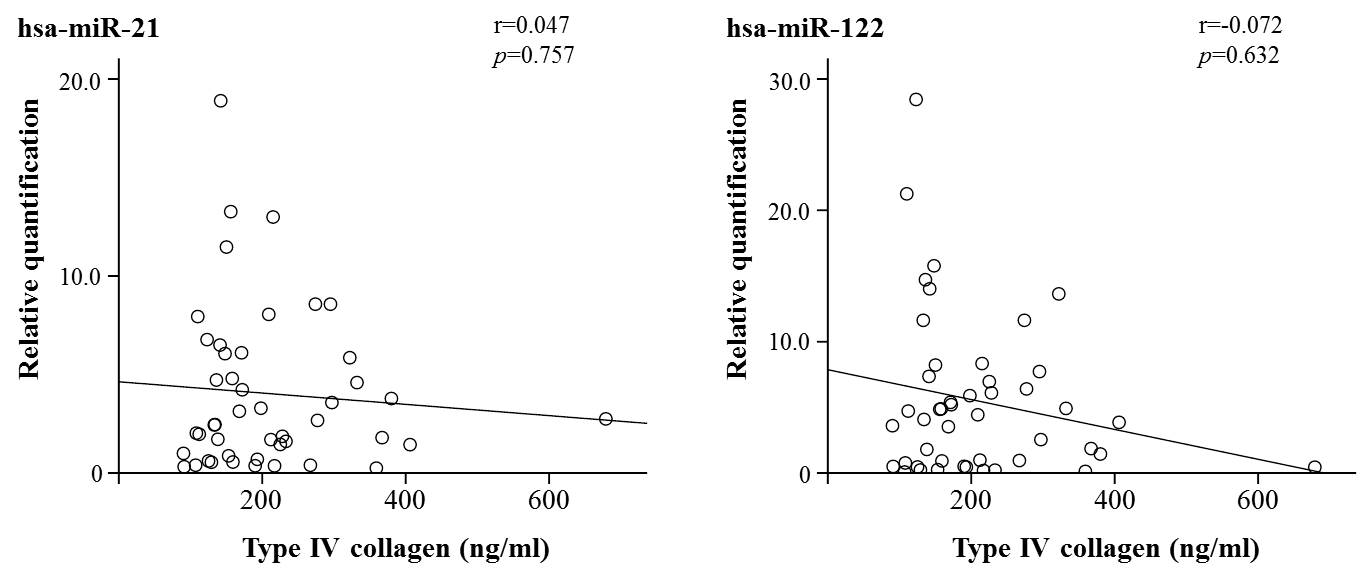

Supplement: S5 Fig — Correlations between serum levels of miR-21or miR-122 and serum type IV collagen levels were determined in patients with AIH. The correlation coefficient was determined by Pearson’s product statistic and the regression line is represented by the solid line. (TIF) [file pone.0136908.s005.tif]

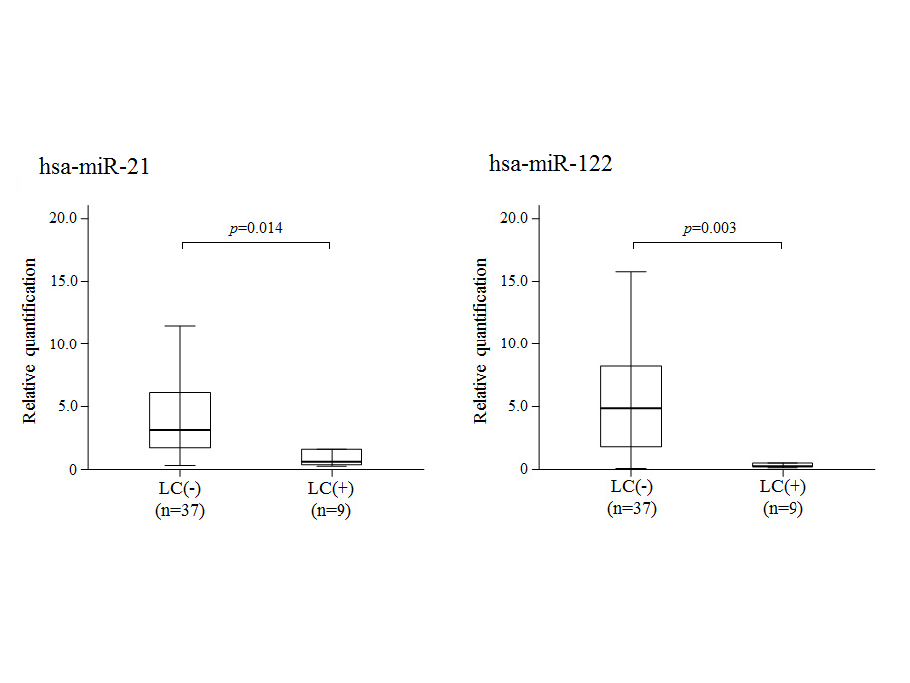

Supplement: S6 Fig — The vertical lines indicate the range, horizontal boundaries of the boxes represent the first and third quartile. Results were compared by non-parametric Mann-Whitney test. (TIFF) [file pone.0136908.s006.tiff]

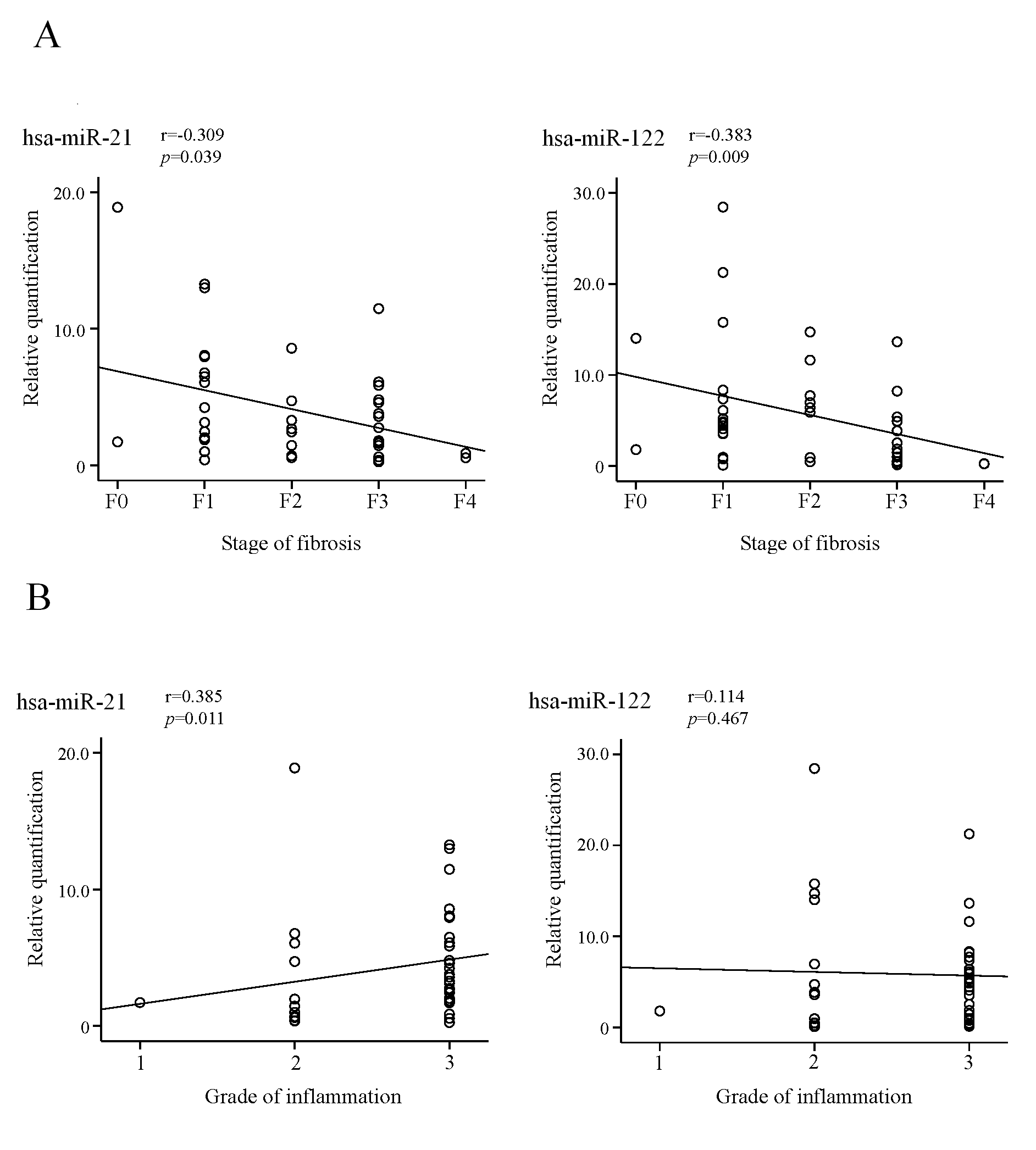

Supplement: S7 Fig — (TIF) [file pone.0136908.s007.tif]
